# Adherence to lifelines diet is associated with lower lung cancer risk in 98,459 participants aged 55 years and above: a large prospective cohort study

**DOI:** 10.3389/fnut.2024.1463481

**Published:** 2024-10-23

**Authors:** Yangpiaoyi Shi, Li Xin, Linglong Peng, Zhiquan Xu, Hang Liu, Qi Wei, Wanhao Tan, Yaxu Wang, Ling Xiang, Haitao Gu

**Affiliations:** ^1^Department of Gastrointestinal Surgery, The Second Affiliated Hospital of Chongqing Medical University, Chongqing, China; ^2^Department of Clinical Nutrition, The Second Affiliated Hospital of Chongqing Medical University, Chongqing, China

**Keywords:** lifeline diet score, lung cancer, diet quality, epidemiology, cohort study, cancer prevention

## Abstract

**Background:**

Lifelines Diet Score (LLDS) was developed based on the 2015 Dutch Dietary Guidelines and current international scientific evidence. As a dietary quality assessment tool, the LLDS aims to evaluate the association between the Lifeline diet and the risk of chronic diseases. However, the evidence linking LLDS to lung cancer risk is currently limited.

**Objective:**

Our objective was to explore whether adherence to the LLDS is associated with reduced incidence and mortality of lung cancer, including its major histological subtypes: small cell lung cancer (SCLC) and non-small cell lung cancer (NSCLC).

**Methods:**

Data for this research were sourced from the Prostate, Lung, Colorectal, and Ovarian (PLCO) Trial. The LLDS for each participant was calculated based on responses to the dietary history questionnaire (DHQ), and subsequently analyzed after being categorized into quintiles. The Cox proportional hazards regression model was utilized to compute the hazard ratios (HRs) and 95% confidence intervals (CIs) for both the incidence and mortality of lung cancer, SCLC and NSCLC. Additionally, stratified analyses were conducted to ascertain possible effect modifiers, and several sensitivity analyses were performed to evaluate the robustness of the findings.

**Results:**

During the mean follow-up periods of 8.8 years for incidence and 15.1 years for mortality, we identified 1,642 new cases and 1,172 related deaths from lung cancer. Participants in the highest quartiles of LLDS compared to those in the lowest exhibited a reduced incidence (HR_Q4:Q1_ = 0.80, 95% CI = 0.68–0.94, *P* for trend = 0.003) and mortality (HR_Q4:Q1_ = 0.81, 95%CI = 0.67–0.98, *P* for trend = 0.009) of lung cancer. Furthermore, this negative association remained for SCLC incidence (HR_Q4:Q1_ = 0.55, 95% CI = 0.35–0.87, *P* for trend = 0.002) and mortality (HR_Q4:Q1_ = 0.42, 95% CI = 0.25–0.70, *P* for trend <0.001). The association between LLDS and the incidence and mortality of lung cancer is not influenced by pre-defined potential effect modifiers (all *P*_interaction_ > 0.05). The sensitivity analyses substantiated the robustness of the results.

**Conclusion:**

In conclusion, our research indicates that among 98,459 U.S. adults aged 55 and older, adherence to the LLDS is linked to a diminished incidence and mortality of lung cancer.

## Introduction

Although the incidence and mortality of lung cancer in the United States have generally declined from 1991 to 2021 due to reductions in smoking, improved disease treatments, and more widespread early cancer screening, it is projected that in 2024, there will be 234,580 newly diagnosed lung cancer cases and an estimated 125,070 deaths (1). Moreover, the incidence of lung cancer is significantly higher among elderly populations compared to younger age groups (2). Therefore, focusing on primary prevention measures for lung cancer within the elderly population in the U.S. is crucial for public health.

Modifiable risk factors play a significant role in the primary prevention of lung cancer. For instance, cessation of smoking has proven effective in reducing lung cancer risk (3). Recent epidemiological studies suggest that, while smoking remains the primary risk factor, certain modifiable dietary factors may also play a role in influencing lung cancer risk (4). Currently supported dietary factors for lung cancer prevention include the intake of fresh fruits, vegetables, nuts, and grains (5–10). However, these studies often focus solely on individual nutrients and overlook the complex interactions among food components (11). Therefore, dietary quality indices constructed from a combination of various dietary factors may better reflect the real-world dietary habits of populations, thus providing more comprehensive dietary guidance for disease prevention (12–14).

The Lifelines Diet Score (LLDS) is a diversified dietary scoring system uniquely developed based on the 2015 Dutch Dietary Guidelines and current international scientific evidence (15). Increasing evidence indicates that this dietary quality has contributed to a reduction in various chronic diseases, making LLDS a valuable tool for assessing dietary quality across different populations in real-world settings (16, 17). In brief, the score ranks and assigns points based on the intake of nine previously validated beneficial foods (including fruit, vegetables, fish, legumes and nuts, whole grains products, oils and soft margarines, coffee, unsweetened dairy, and tea), and three detrimental foods (butter and hard margarines, red and processed meat, sugar-sweetened beverages) (15). A higher intake of beneficial foods results in higher scores, while a lower intake of detrimental foods also contributes positively to the score. Emerging research suggests that adherence to the LLDS not only reduces the risk of chronic illnesses including asthma, chronic kidney disease, and inflammatory bowel disease but also decreases mortality risk among populations with cardiovascular metabolic diseases (16–19). However, research exploring the relationship between the LLDS and cancer risk remains relatively scarce. Until now, only a limited number of studies have concerned this issue. For example, a prospective cohort study found no significant association between the LLDS and the risk of gastrointestinal cancer (20). Conversely, a case–control study demonstrated that higher LLDS was related to a reduced risk of breast cancer (21). To our knowledge, there have been no studies specifically investigating the potential association between the LLDS and lung cancer risk yet. To address this knowledge gap, we conducted a prospective analysis of U.S. adults aged 55 years and older based on the Prostate, Lung, Colorectal, and Ovarian (PLCO) cohort.

## Methods

### Study design and population

The study population is derived from the PLCO Trial, a multicenter clinical trial aimed at assessing whether specific screening tests can reduce cancer mortality. The detailed trial methodology has been extensively described elsewhere (22). In summary, eligible participants were recruited from 10 study centers across the U.S. in the period 1993–2001, all of whom provided informed consent. Upon randomization, participants were evenly allocated to the control group received standard medicine care, and the intervention group underwent regular cancer screening as per the PLCO protocol. At the onset of the PLCO trial, each participant completed a baseline questionnaire (BQ), voluntarily reporting baseline information such as demographic characteristics and medical history. In 1998, the PLCO screening centers introduced a dietary history questionnaire (DHQ), which was to be completed concurrently with the BQ for participants enrolled after 1998, and for those enrolled before 1998, they were required to supplement any previously incomplete DHQ. The DHQ comprised inquiries about the intake of 124 foods and supplements over the past year, including intake amount, daily frequency, and other details. Participants were followed up upon entry into the PLCO trial, with data collection including cancer diagnoses up to 2009 and mortality status up to 2018. This study has received approval from the National Cancer Institute (NCI) (Project ID: PLCO-1560).

In our study, we further excluded: (1) individuals who did not return or complete the BQ (*n* = 4,918); (2) individuals with invalid DHQ completion, defined as lacking completion date, confirmed death before completing, having ≥8 missing responses, or extreme calorie intake values (top, or bottom 1%) (*n* = 38,462); (3) individuals with any type of personal history of cancer before entering the DHQ (*n* = 9,684); (4) individuals who experienced outcome events between entering the DHQ and DHQ completion, including the occurrence of lung cancer, death, or loss to follow-up (*n* = 68); (5) individuals with extreme energy intake (less than 800 kcal or more than 4,200 kcal for males, and less than 600 kcal or more than 3,500 kcal for females) (*n* = 3,296). Ultimately, 98,459 individuals were incorporated in this research. The exclusion and inclusion process are depicted in the Supplementary Figure S1.

### Collection of dietary data and covariate assessment

Baseline information of participants was collected from the BQ, including sex, race, marital status, education level, body mass index (BMI), pack-years, history of respiratory diseases, history of diabetes, history of hypertension, history of aspirin use, family history of lung cancer, cigarette smoking status, and randomization arm. Body mass index was calculated as weight (kg) divided by height (m) squared. Respiratory diseases included bronchitis and emphysema. Dietary data, age and alcohol drinking status were collected using the DHQ, whose validity has been confirmed through the United States National Health and Nutrition Examination Survey (23). The DHQ includes the 2015 Healthy Eating Index (HEI-2015), which is used to measure individual dietary quality (13). In this study, the HEI-2015 was used as a benchmark to compare the effectiveness and value of the LLDS in measuring dietary quality.

### Calculation of LLDS

The LLDS is developed based on the 2015 Dutch Dietary Guidelines and international evidence on the relationship between diet and chronic disease (15). Specifically, the 110 items in the Food Frequency Questionnaire (FFQ) are divided into 22 food groups, which are categorized into four classes based on their impact on human health: positive, negative, neutral, and unknown. Ultimately, positive groups (including fruit, vegetables, fish, legumes and nuts, whole grains products, oils and soft margarines, coffee, unsweetened dairy, and tea) and negative groups (butter and hard margarines, red and processed meat, sugar-sweetened beverages) were selected for inclusion in the scoring scheme. Food intake was normalized to a standard energy intake of 1,000 kcal. The energy-adjusted food intake was then distributed into quintiles ranging from 0 to 4. The highest quintile was assigned a score of 4 for positive food groups, while the lowest quintile was assigned a score of 0. In contrast, the scoring methodology for negative food groups was inverted. After all, the scores for all food groups were aggregated to derive the LLDS. A higher score indicates greater adherence to LLDS, reflecting higher dietary quality (15).

### Outcome ascertainment

Each participant received an annual report requesting information on any cancer diagnoses. In the event of a diagnosis report, they were asked to provide the diagnosis date, site, cancer type, the medical institution making the diagnosis, and contact information for the diagnosing physician. For non-responses, the research team reached out to participants via email or phone to verify their cancer diagnosis and survival status. For each case of lung cancer, researchers made efforts to confirm the diagnosis and obtain detailed information by contacting the diagnosing facility or physician. To ensure comprehensive mortality information, the trial also regularly checked national death registries and used the International Classification of Diseases, Ninth Revision (ICD-9), based on death certificates, to ascertain causes of death.

### Statistical analysis

This study encountered missing data for several covariates. Among them, smoking pack-years, a continuous variable, had the largest proportion of missing data at 1.12%. Other covariates had missing values for <1% of participants (Supplementary Table S1). Therefore, we imputed missing values of continuous variables using median imputation, including BMI and pack-years. For categorical variables, including race, marital status, education level, smoking status, family history of lung cancer, history of emphysema, history of bronchitis, history of hypertension, history of diabetes, and aspirin use, the missing values were imputed using mode imputation. For mode imputation, we first identified the most frequently occurring category (i.e., the mode) for each variable, and then used this mode to fill in the missing values for the corresponding variable (24). After imputation, the distribution of categorical variables and the means of continuous variables remained virtually unchanged compared to the pre-imputation values (Supplementary Table S1).

Participants were grouped into quartiles depending on their LLDS. Cox proportional hazards regression model was employed, with follow-up time as the time variable. The lowest quartile is considered as the reference group. Hazard ratios (HRs) and 95% confidence intervals (CIs) were computed for each quartile relative to the reference group. As shown in Supplementary Figure S2. The follow-up data regarding the incidence of lung cancer spanned from the completion date of the DHQ to the occurrences of lung cancer diagnosis, loss to follow-up, or termination of follow-up (12/31/2009). For lung cancer mortality, the follow-up period concluded in 2018. The median LLDS was allocated to participants in each quartile and treated as a continuous variable in Cox regression analysis to derive a trend *p*-value, evaluating the statistical significance. Furthermore, LLDS was analyzed as a continuous variable to assess the risk estimate associated with 1-point increment in LLDS. Based on existing medical knowledge, clinical experience, and a review of relevant previous studies, we constructed three models: unadjusted model, Model 1 adjusted for basic demographic characteristics, and Model 2 as a fully adjusted model. Specially, Model 1 adjusted for age, sex, race, marital status, and education level. As the fully adjusted model, Model 2 not only adjusted for the variables in Model 1 but also additionally adjusted for BMI, alcohol drinking status, cigarette smoking status, pack-years, history of respiratory diseases, history of diabetes, history of hypertension, history of aspirin use, family history of lung cancer, and randomization arm. In addition, we also used restricted cubic spline model with three knots at the 10th, 50th, and 90th centiles to flexibly model the relationship between the overall LLDS and the incidence and mortality of lung cancer. The *p-*value for non-linearity was calculated by testing whether the regression coefficient of the second spline was equal to zero. Meanwhile, using the same methodology, we also examined the association between LLDS and specific lung cancer subtypes, including NSCLC and SCLC. Predetermined variables representing potential confounding factors was incorporated for conducting stratified analyses, including age (>65 years and ≤65 years), sex (male and female), smoking status (No and Current/former), family history of lung cancer (No and Yes/Possibly), BMI at baseline (>30 kg/m^2^ and ≤30 kg/m^2^), aspirin use regularly (No and Yes), history of emphysema (No and Yes), history of bronchitis (No 11and Yes), and trail arm (Intervention and Control). Given that smoking is a significant risk factor for lung cancer, and smoking cessation can substantially reduce the risk of lung cancer mortality (25), we further categorized smoking status into three groups: never, current, and former. This classification enabled us to conduct a more in-depth investigation of the interaction effect of smoking on the association between LLDS and lung cancer. The significance of the multiplicative interaction between the above stratification factors and LLDS was examined using likelihood ratio tests.

Finally, a sequence of sensitivity analyses were conducted to reinforce the stability of the results: (1) exclusion of individuals with extreme BMI (top 1% and bottom 1%); (2) exclusion of individuals who experienced the outcome (including incidence and mortality of lung cancer) within the initial 2 or 4 years of follow-up, which may have partially mitigated the potential for reverse causality effects; (3) exclusion of individuals with respiratory comorbidities such as emphysema and chronic bronchitis, as these conditions may increase the risk of lung cancer (26); (4) adjustment for daily cigarette consumption (0, 1–20, or >20) instead of pack-years, to enhance the statistical power of the analysis; (5) excluded participants with missing data to determine whether the obtained results were influenced by the imputation of missing data; (6) HEI-2015 scores was used in place of LLDS for calculations to test the effectiveness of LLDS in assessing dietary quality.

All statistical analyses were performed using R software version 4.3.2, with a significance level set at *p* < 0.05.

## Results

### Baseline characteristics

This study encompassed 98,459 participants, with an average age of 65.5 years (standard deviation of 5.7 years). Participants were stratified based on their LLDS into four quartiles: Quartile 1 (0–19), Quartile 2 (20–24), Quartile 3 (25–29), and Quartile 4 (30–45), as detailed in Table 1. Those in the highest LLDS quartile typically exhibited characteristics such as a higher likelihood of being female, possessing greater educational levels, and a lower prevalence of smoking. Additionally, they had lower BMI and reduced energy intake. Regarding medical histories, the highest quartile had fewer instances of diabetes, hypertension, and emphysema compared to the lowest quartile. Notably, the increase in LLDS quartiles corresponded with higher HEI-2015 scores, validating LLDS as an effective measure of dietary quality against the widely recognized HEI-2015 standard.

**Table 1:** Baseline characteristics of study population according to quartiles of LLDS.

		Quartiles of overall LLDS			
Characteristics	Overall	Quartile 1 (0–19)	Quartile 2 (20–24)	Quartile 3 (25–29)	Quartile 4 (30–45)
Number of participants	98,459	24,708	27,245	26,066	20,440
LLDS	24.0 ± 6.5	15.7 ± 3.0	22.1 ± 1.4	26.9 ± 1.4	37.4 ± 3.0
Age	65.5 ± 5.7	64.8 ± 5.7	65.4 ± 5.7	65.8 ± 5.8	66.1 ± 5.7
Sex					
Male	47,218 (48.0%)	16,423 (66.5%)	14,387 (52.8%)	10,466 (40.2%)	5,942 (29.1%)
Female	51,241 (52.0%)	8,285 (33.5%)	12,858 (47.2%)	15,600 (59.8%)	14,498 (70.9%)
Marital					
Married	77,374 (78.6%)	19,839 (80.3%)	21,712 (79.7%)	20,328 (78.0%)	15,495 (75.8%)
Unmarried	21,085 (21.4%)	4,869 (19.7%)	5,533 (20.3%)	5,738 (22.0%)	4,945 (24.2%)
Race					
White	91,221 (92.6%)	23,376 (94.6%)	25,491 (93.6%)	23,975 (92.0%)	18,379 (89.9%)
Non-white	7,238 (7.4%)	1,332 (5.4%)	1754 (6.4%)	2091 (8.0%)	2061 (10.1%)
Education					
College below	62,599 (63.6%)	17,435 (70.6%)	17,580 (64.5%)	16,056 (61.6%)	11,528 (56.4%)
College graduate	17,353 (17.6%)	3,899 (15.8%)	4,817 (17.7%)	4,711 (18.1%)	3,926 (19.2%)
Postgraduate	18,507 (18.8%)	3,374 (13.7%)	4,848 (17.8%)	5,299 (20.3%)	4,986 (24.4%)
BMI at baseline (kg/m2)	27.2 ± 4.8	28.0 ± 4.8	27.5 ± 4.8	26.9 ± 4.7	26.1 ± 4.6
Alcohol drinking status					
No	26,681 (27.1%)	7,239 (29.3%)	7,301 (26.8%)	6,909 (26.5%)	5,232 (25.6%)
Yes	71,778 (72.9%)	17,469 (70.7%)	19,944 (73.2%)	19,157 (73.5%)	15,208 (74.4%)
Smoking status					
No	47,233 (48.0%)	10,463 (42.3%)	12,584 (46.2%)	13,177 (50.6%)	11,009 (53.9%)
Current/Former	51,226 (52.0%)	14,245 (57.7%)	14,661 (53.8%)	12,889 (49.4%)	9,431 (46.1%)
Smoking pack-years	17.5 ± 26.4	22.7 ± 30.3	18.6 ± 27.2	15.4 ± 24.2	12.4 ± 21.1
Family history of lung cancer					
No	85,845 (87.2%)	21,388 (86.6%)	23,734 (87.1%)	22,803 (87.5%)	17,920 (87.7%)
Yes	10,266 (10.4%)	2,548 (10.3%)	2,853 (10.5%)	2,718 (10.4%)	2,147 (10.5%)
Possibly	2,348 (2.4%)	772 (3.1%)	658 (2.4%)	545 (2.1%)	373 (1.8%)
Emphysema history					
No	96,410 (97.9%)	24,031 (97.3%)	26,649 (97.8%)	25,570 (98.1%)	20,160 (98.6%)
Yes	2049 (2.1%)	677 (2.7%)	596 (2.2%)	496 (1.9%)	280 (1.4%)
Chronic bronchitis history					
No	94,278 (95.8%)	23,650 (95.7%)	26,067 (95.7%)	24,961 (95.8%)	19,600 (95.9%)
Yes	4,181 (4.2%)	1,058 (4.3%)	1,178 (4.3%)	1,105 (4.2%)	840 (4.1%)
History of hypertension					
No	66,641 (67.7%)	16,397 (66.4%)	18,261 (67.0%)	17,627 (67.6%)	14,356 (70.2%)
Yes	31,818 (32.3%)	8,311 (33.6%)	8,984 (33.0%)	8,439 (32.4%)	6,084 (29.8%)
Aspirin use					
No	52,242 (53.1%)	13,186 (53.4%)	14,242 (52.3%)	13,809 (53.0%)	11,005 (53.8%)
Yes	46,217 (46.9%)	11,522 (46.6%)	13,003 (47.7%)	12,257 (47.0%)	9,435 (46.2%)
History of diabetes					
No	91,990 (93.4%)	22,923 (92.8%)	25,362 (93.1%)	24,363 (93.5%)	19,342 (94.6%)
Yes	6,469 (6.6%)	1785 (7.2%)	1883 (6.9%)	1703 (6.5%)	1,098 (5.4%)
Randomized arms					
Intervention	50,151 (50.9%)	12,553 (50.8%)	13,829 (50.8%)	13,272 (50.9%)	10,497 (51.4%)
Control	48,308 (49.1%)	12,155 (49.2%)	13,416 (49.2%)	12,794 (49.1%)	9,943 (48.6%)
HEI-2015 scores	66.6 ± 9.7	58.0 ± 8.5	64.9 ± 7.7	69.9 ± 7.0	75.2 ± 6.3
Energy intake from diet (kcal/day)	1728.7 ± 658.0	1925.1 ± 720.1	1758.8 ± 662.5	1637.8 ± 608.2	1567.3 ± 563.8
Positive food consumption					
Vegetable (g/day)	284.8 ± 181.9	217.9 ± 138.0	263.8 ± 164.2	303.0 ± 181.5	370.6 ± 211.2
Fruit (g/day)	275.2 ± 213.3	194.7 ± 181.1	253.0 ± 198.5	302.1 ± 212.6	368.0 ± 226.6
Legume and Nuts (g/day)	24.0 ± 29.5	15.5 ± 19.9	21.3 ± 25.2	25.9 ± 29.9	35.5 ± 38.5
Whole grain product (g/day)	61.5 ± 59.7	41.5 ± 46.4	56.1 ± 54.4	67.7 ± 61.1	85.2 ± 68.7
Fish (g/day)	15.5 ± 18.3	11.1 ± 14.1	14.2 ± 17.0	16.6 ± 19.1	21.2 ± 21.8
Tea (g/day)	264.7 ± 469.6	155.1 ± 401.5	237.3 ± 455.0	297.2 ± 479.5	392.3 ± 515.0
Coffee (g/day)	846.1 ± 791.8	802.3 ± 846.9	874.0 ± 817.0	864.5 ± 770.9	838.5 ± 708.1
Oil and soft margarine (g/day)	1.7 ± 3.4	1.0 ± 2.6	1.5 ± 3.2	1.8 ± 3.4	2.4 ± 4.0
Dairies unsweetened (g/day)	51.7 ± 115.5	41.9 ± 128.7	47.7 ± 115.7	51.5 ± 107.2	69.2 ± 106.0
Negative food consumption					
Red and processed meat (g/day)	12.3 ± 14.6	20.0 ± 19.4	13.7 ± 14.0	9.2 ± 10.3	5.0 ± 6.3
Butter and hard margarine (g/day)	2.7 ± 5.4	4.7 ± 7.2	3.0 ± 5.4	1.9 ± 4.1	1.0 ± 2.7
Beverages (g/day)	217.4 ± 389.9	399.9 ± 545.1	223.0 ± 365.5	146.0 ± 275.3	80.5 ± 181.4

Descriptive statistics are presented as (mean ± standard deviation) and number (percentage) for continuous and categorical.

### The relationship between LLDS and lung cancer incidence

**Figure 1:**
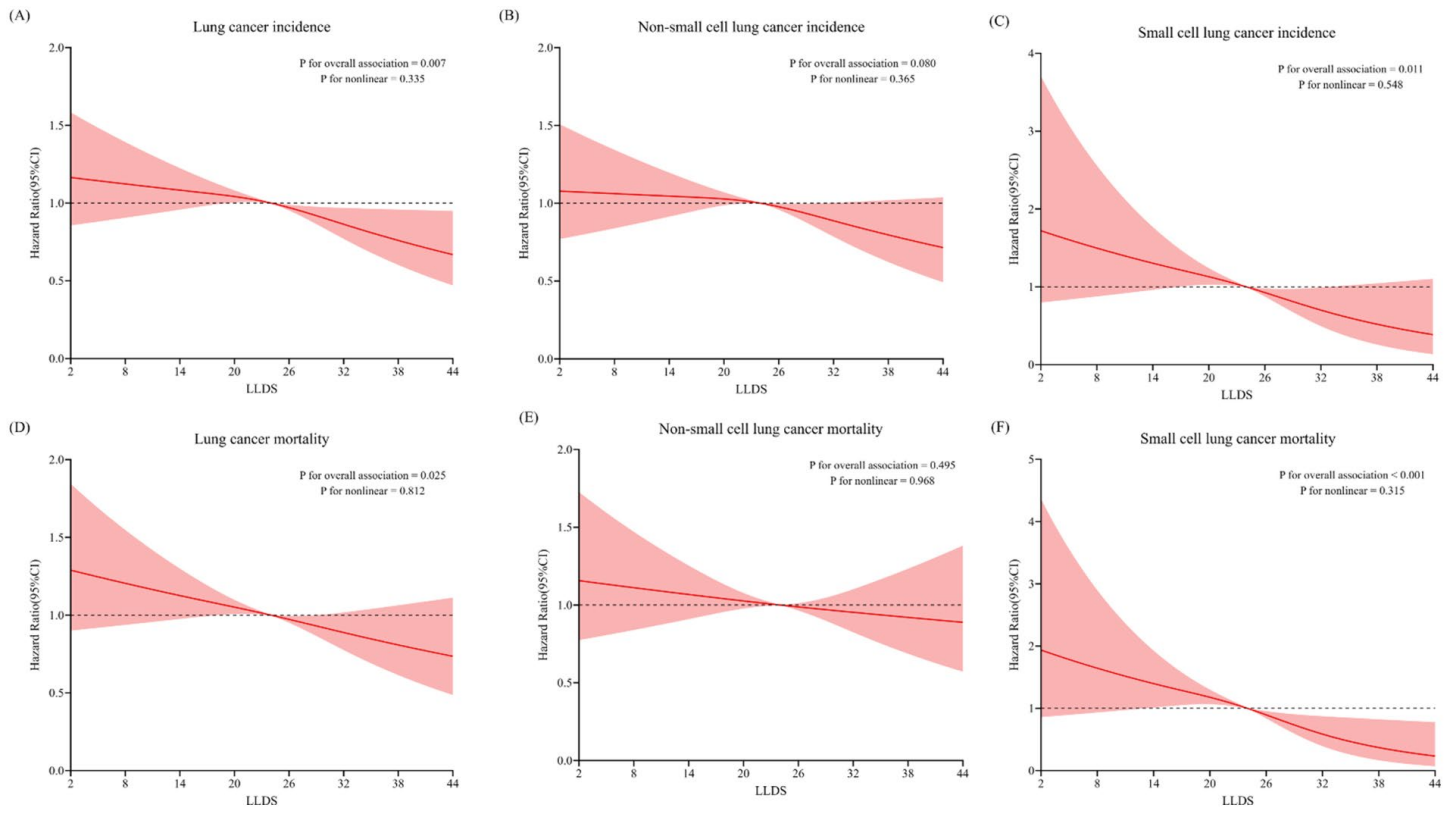
Non-linear Dose–response analysis on the association of LLDS and the incidence and mortality of lung cancer, as well as its major histological subtypes: SCLC and NSCLC (A/D: all lung cancer; B/E: NSCLC; C/F: SCLC).

During the mean follow-up period of 8.8 years, a total of 1,642 new lung cancer cases were diagnosed, with an incidence of 0.19 cases per 100 person-years. Among the confirmed cases, there were 1,408 instances of NSCLC and 234 cases of SCLC. In the fully adjusted multivariable model, individuals in the highest quartile of LLDS demonstrated a significantly lower incidence of lung cancer compared to those in the lowest quartile. HRs indicated inverse relationships across different types of lung cancer: for overall lung cancer, the HR_Q4:Q1_ was 0.80 (95% CI = 0.68–0.94, *P* for trend = 0.003), and for SCLC, the HR_Q4:Q1_ was 0.55 (95% CI = 0.35–0.87, *P* for trend = 0.002). Although the HR for NSCLC indicated a reduced risk at 0.85 (95% CI = 0.72–1.01), this association did not achieve statistical significance (*P* for trend = 0.063) (Table 2). When LLDS is analyzed as a continuous variable, a 1-point increment in LLDS was associated with a 1% decrease in the incidence of overall lung cancer, with a specific reduction of 3% for SCLC. Further analysis using restricted cubic spline models revealed an inverse linear dose–response relationship between the full LLDS and the incidence of lung cancer (*P* for non-linearity = 0.335) and SCLC (*P* for non-linearity = 0.548), as depicted in Figure 1.

**Table 2:** Hazard ratios of the association between LLDS and lung cancer incidence as well as its subtypes.

Quartiles of LLDS	Cases	Person-years	Incidence rate per 100 person-years (95% confidence interval)	Hazard ratio (95% confidence interval) by LLDS		
				Unadjusted	Model 1^a^	Model 2^b^
Lung cancer						
Quartile 1	518	214401.6	0.24 (0.22, 0.26)	1.00 (Reference)	1.00 (Reference)	1.00 (Reference)
Quartile 2	485	240081.7	0.20 (0.18, 0.22)	0.83 (0.74, 0.94)	0.88 (0.78, 1.00)	0.95 (0.84, 1.07)
Quartile 3	390	231673.8	0.17 (0.15, 0.19)	0.69 (0.61, 0.79)	0.77 (0.67, 0.88)	0.87 (0.76, 1.00)
Quartile 4	249	183650.8	0.14 (0.12, 0.15)	0.56 (0.48, 0.65)	0.66 (0.57, 0.78)	0.80 (0.68, 0.94)
*P* for trend				<0.001	<0.001	0.003
1-point increment in LLDS	1,642	869807.9	0.19 (0.18, 0.20)	0.97 (0.96, 0.98)	0.98 (0.97, 0.99)	0.99 (0.98, 1.00)
Non-small cell lung cancer						
Quartile 1	429	214401.6	0.20 (0.18, 0.22)	1.00 (Reference)	1.00 (Reference)	1.00 (Reference)
Quartile 2	412	240081.7	0.17 (0.16, 0.19)	0.86 (0.75, 0.98)	0.91 (0.79, 1.04)	0.97 (0.85, 1.11)
Quartile 3	345	231673.8	0.15 (0.13, 0.17)	0.74 (0.64, 0.85)	0.82 (0.71, 0.95)	0.93 (0.80, 1.07)
Quartile 4	222	183650.8	0.12 (0.11, 0.14)	0.60 (0.51, 0.71)	0.71 (0.60, 0.84)	0.85 (0.72, 1.01)
*P* for trend				<0.001	<0.001	0.063
1-point increment in LLDS	1,408	869807.9	0.16 (0.15, 0.17)	0.97 (0.96, 0.98)	0.98 (0.97, 0.99)	0.99 (0.98, 1.00)
Small cell lung cancer						
Quartile 1	89	214401.6	0.04 (0.03, 0.05)	1.00 (Reference)	1.00 (Reference)	1.00 (Reference)
Quartile 2	73	240081.7	0.03 (0.02, 0.04)	0.73 (0.54, 1.00)	0.78 (0.57, 1.07)	0.84 (0.61, 1.15)
Quartile 3	45	231673.8	0.02 (0.01, 0.03)	0.47 (0.33, 0.67)	0.52 (0.36, 0.75)	0.62 (0.43, 0.90)
Quartile 4	27	183650.8	0.01 (0.01, 0.02)	0.35 (0.23, 0.54)	0.42 (0.27, 0.66)	0.55 (0.35, 0.87)
*P* for trend				<0.001	<0.001	0.002
1-point increment in LLDS	234	869807.9	0.03 (0.02, 0.03)	0.95 (0.93, 0.96)	0.95 (0.93, 0.98)	0.97 (0.95, 0.99)

^a^Model 1 was controlled with age (continuous), sex (male, female), race (white, non-white), education levels (college below, college graduate, postgraduate) and marital status (no, yes). ^b^Model2 was additionally controlled with smoking status (never, current/former), pack-years (continuous), alcohol drinking status (no, yes), BMI (continuous), randomization arm (intervention group, control group), family history of lung cancer (no, yes, possibly), history of hypertension (no, yes), history of diabetes (no, yes), history of chronic bronchitis (no, yes), history of emphysema (no, yes) and aspirin use (no, yes).

### The relationship between LLDS and lung cancer mortality

Over a 15.1-year follow-up, the study documented 1,172 lung cancer-related deaths, comprising 967 NSCLC and 205 SCLC cases. Multivariable analyses revealed that individuals in the highest LLDS quartile had significantly lower mortality for both overall lung cancer (HR_Q4:Q1_ = 0.81, 95% CI = 0.67–0.98, *P* for trend = 0.009) and SCLC (HR_Q4:Q1_ = 0.42, 95% CI = 0.25–0.70, *P* for trend <0.001), as detailed in Table 3. No significant effect on NSCLC mortality was observed (HR_Q4:Q1_ = 0.91, 95% CI = 0.74–1.11, *P* for trend = 0.268). For 1-point increment in LLDS, there was a corresponding 1% reduction in overall lung cancer mortality and a 4% decrease in SCLC mortality. Restricted cubic spline analysis confirmed an inverse linear dose–response relationship between LLDS and mortality for both overall lung cancer and SCLC, with respective *P* for non-linearity values of 0.812 and 0.315, as depicted in Figure 1.

**Table 3:** Hazard ratios of the association between LLDS and lung cancer mortality as well as its subtypes.

Quartiles of LLDS	Cases	Person-years	Incidence rate per 100 person-years (95% confidence interval)	Hazard ratio (95% confidence interval) by LLDS		
				Unadjusted	Model 1^a^	Model 2^b^
Lung cancer						
Quartile 1	382	360522.4	0.11 (0.10, 0.12)	1.00 (Reference)	1.00 (Reference)	1.00 (Reference)
Quartile 2	343	408370.2	0.08 (0.08, 0.09)	0.80 (0.69, 0.93)	0.86 (0.74, 1.00)	0.92 (0.79, 1.06)
Quartile 3	268	397223.4	0.07 (0.06, 0.08)	0.65 (0.55, 0.76)	0.73 (0.62, 0.86)	0.83 (0.71, 0.98)
Quartile 4	179	320693.4	0.06 (0.05, 0.06)	0.54 (0.45, 0.65)	0.67 (0.55, 0.80)	0.81 (0.67, 0.98)
*P* for trend				<0.001	<0.001	0.009
1-point increment in LLDS	1,172	1486809.4	0.08 (0.07, 0.08)	0.97 (0.96, 0.97)	0.98 (0.97, 0.99)	0.99 (0.98, 1.00)
Non-small cell lung cancer						
Quartile 1	318	360522.4	0.08 (0.07, 0.09)	1.00 (Reference)	1.00 (Reference)	1.00 (Reference)
Quartile 2	266	408370.2	0.07 (0.06, 0.08)	0.82 (0.70, 0.97)	0.88 (0.75, 1.04)	0.94 (0.80, 1.11)
Quartile 3	213	397223.4	0.06 (0.05, 0.07)	0.71 (0.59, 0.84)	0.81 (0.68, 0.96)	0.91 (0.76, 1.08)
Quartile 4	170	320693.4	0.05 (0.04, 0.06)	0.61 (0.50, 0.74)	0.76 (0.62, 0.93)	0.91 (0.74, 1.11)
*P* for trend				<0.001	0.003	0.268
1-point increment in LLDS	967	1486809.4	0.07 (0.06, 0.07)	0.97 (0.96, 0.98)	0.98 (0.97, 0.99)	0.99 (0.98, 1.00)
Small cell lung cancer						
Quartile 1	81	360522.4	0.02 (0.02, 0.03)	1.00 (Reference)	1.00 (Reference)	1.00 (Reference)
Quartile 2	66	408370.2	0.02 (0.01, 0.02)	0.73 (0.53, 1.01)	0.76 (0.55, 1.06)	0.82 (0.59, 1.14)
Quartile 3	38	397223.4	0.01 (0.01, 0.01)	0.44 (0.30, 0.64)	0.47 (0.31, 0.69)	0.55 (0.37, 0.82)
Quartile 4	20	320693.4	0.01 (0.00, 0.01)	0.29 (0.18, 0.47)	0.33 (0.20, 0.54)	0.42 (0.25, 0.70)
*P* for trend				<0.001	<0.001	<0.001
1-point increment in LLDS	205	1486809.4	0.01 (0.01, 0.02)	0.94 (0.92, 0.96)	0.94 (0.92, 0.97)	0.96 (0.93, 0.98)

^a^Model 1 was controlled with age (continuous), sex (male, female), race (white, non-white), education levels (college below, college graduate, postgraduate) and marital status (no, yes). ^b^Model 2 was additionally controlled with smoking status (never, current/former), pack-years (continuous), alcohol drinking status (no, yes), BMI (continuous), randomization arm (intervention group, control group), family history of lung cancer (no, yes, possibly), history of hypertension (no, yes), history of diabetes (no, yes), history of chronic bronchitis (no, yes), history of emphysema (no, yes) and aspirin use (no, yes).

### Additional analyses

**Figure 2:**
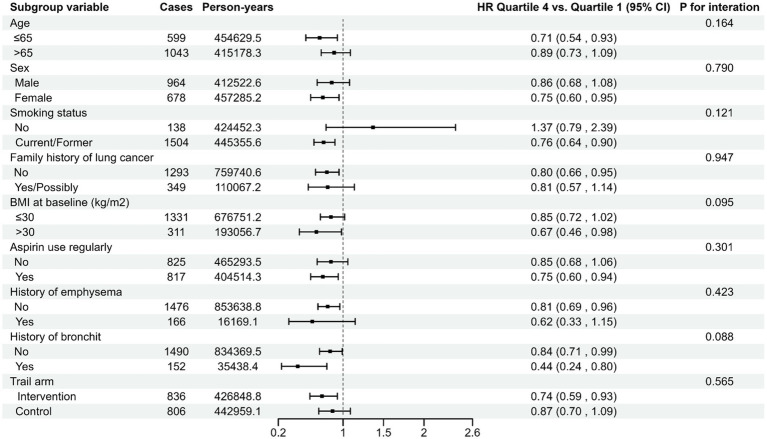
Stratified analyses on the associations of LLDS and lung cancer incidence. For LLDS, hazard ratios were adjusted for age (continuous), sex (male, female), race (white, non-white), education levels (college below, college graduate, postgraduate), marital status (no, yes), smoking status (never, current/former), pack-years (continuous), alcohol drinking status (no, yes), BMI (continuous), randomization arm (intervention group, control group), family history of lung cancer (no, yes, possibly), history of hypertension (no, yes), history of diabetes (no, yes), history of chronic bronchitis (no, yes), history of emphysema (no, yes) and aspirin use (no, yes).

**Figure 3:**
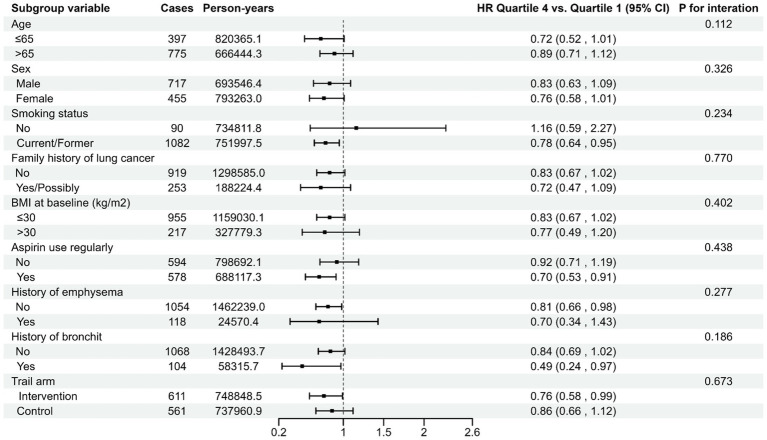
Stratified analyses on the associations of LLDS and lung cancer mortality. For LLDS, hazard ratios were adjusted for age (continuous), sex (male, female), race (white, non-white), education levels (college below, college graduate, postgraduate), marital status (no, yes), smoking status (never, current/former), pack-years (continuous), alcohol drinking status (no, yes), BMI (continuous), randomization arm (intervention group, control group), family history of lung cancer (no, yes, possibly), history of hypertension (no, yes), history of diabetes (no, yes), history of chronic bronchitis (no, yes), history of emphysema (no, yes) and aspirin use (no, yes).

The association between the LLDS and both the incidence and mortality of lung cancer is not affected by pre-defined potential effect modifiers (all *P*_interaction_ > 0.05) (Figures 2, 3). When we conducted subgroup analyses using a more refined classification of smoking status (never, current, and former smokers), this inverse relationship is not affected by the refined smoking status (*P*_interaction_ > 0.05) (Supplementary Table S5). The sensitivity analyses conducted reinforced the robustness of the association between LLDS and lung cancer outcomes (Supplementary Tables S2, S3). Key adjustments included the exclusion of individuals with extreme BMI values and those who experienced the outcome within the first 2–4 years of follow-up. Additionally, excluding participants with respiratory comorbidities such as emphysema and chronic bronchitis did not alter the results. Further adjustments for daily cigarette consumption instead of pack-years, and excluded participants with missing data, also supported the original findings. To further validate the effectiveness of LLDS as a measure of dietary quality, we substituted LLDS with the widely validated dietary assessment tool, HEI-2015. The results still demonstrated a relationship with higher HEI-2015 and decreased lung cancer incidence and mortality. This comparison underscores the reliability of LLDS in assessing dietary quality and its relationship with lung cancer incidence and mortality.

## Discussion

The results of our study suggest that higher adherence to LLDS was associated with lower incidence and mortality of lung cancer. Notably, a comparable association was observed in the case of SCLC. The restricted cubic spline models demonstrate an inverse linear dose–response association between the entire LLDS and both the incidence and mortality of lung cancer, as well as SCLC. Finally, the preliminary results maintained their robustness throughout the sensitivity analyses.

The Mediterranean diet, Dietary Approaches to Stop Hypertension (DASH), and LLDS are recognized as healthy dietary patterns. They advocate for an increased consumption of fish and plant-based foods such as vegetables, fruits, whole grains, legumes, nuts, and seeds, while restricting red and processed meat. However, these dietary patterns differ in certain specific components. The Mediterranean diet emphasizes the use of olive oil, moderate consumption of red wine, and relies on fish as the primary source of protein. In contrast, the DASH diet discourages alcohol consumption, emphasizes low-fat or fat-free dairy products, and strictly limits sodium intake. The LLDS uniquely encourages the consumption of coffee and tea. In fact, the Mediterranean diet and DASH diet have been demonstrated to reduce the lung cancer risk. For instance, a meta-analysis comprising eight cohort studies and one case–control study revealed that adherence to the Mediterranean diet is related to a decreased risk of lung cancer (27). Furthermore, a prospective cohort study conducted by our team found that higher adherence to the DASH is significantly related with a decreased lung cancer incidence (28). While these three healthy dietary patterns share similarities in their dietary composition, their formation backgrounds and objectives differ. The Mediterranean diet originates from the traditional lifestyle practices of populations residing along the Mediterranean coast (29), whereas the DASH diet is a carefully designed dietary regimen aimed at reducing blood pressure (12). In contrast, LLDS is developed based on the 2015 Dutch dietary guidelines and contemporary international scientific evidence concerning the association between dietary habits and 10 chronic diseases, rather than being structured around cultural traditions or tailored to specific diseases (15). This endows LLDS with broader applicability, an international perspective, and significant scientific validity and reliability.

At present, a plethora of epidemiological studies have provided evidence supporting the significant decreased risk of chronic diseases connected with higher LLDS. For instance, a case–control study conducted by Sohouli et al. (21), which revealed that a higher LLDS was significantly connected with a decreased risk of breast cancer (OR: 0.21; 95% CI: 0.11–0.43; *P* trend <0.001). Additionally, a prospective study conducted within the Lifeline cohort revealed that individuals in the uppermost quartile of LLDS had a 17% decrease in the risk of chronic kidney disease (16). Another large prospective study revealed that adherence to LLDS was linked to a decreased incidence of inflammatory bowel disease (OR: 0.95, 95% CI: 0.92–0.99, *P* trend = 0.009) (17). Concurrently, results from a study in the Lifelines Cohort revealed that elevated LLDS were associated with reduced all-cause mortality (19). In summary, LLDS has been validated to confer beneficial effects on human health. However, existing literature does not provide evidence supporting the effectiveness of LLDS in reducing the risk of lung cancer. Therefore, we conducted a prospective analysis of U.S. adults aged 55 years and older for assessing the relationship between LLDS and lung cancer risk.

The subsequent mechanisms potentially elucidate why adherence to LLDS may mitigate the risk of lung cancer. Firstly, LLDS prioritizes the intake of a plant-based diet, encompassing foods such as fruits, vegetables, grains, nuts, and seeds, which are abundant in dietary fiber. Insufficient dietary fiber intake in the human body may result in impairment of the intestinal mucosal barrier and dysbiosis of the gut microbiota (30), potentially facilitating the ingress of pathogenic microorganisms and perturbation of the internal microbiota, including the pulmonary microbiota (31). The enzymatic mechanism of the intestinal microbiota facilitates the absorption of dietary fiber, producing short-chain fatty acids. These compounds subsequently modulate the functions of immune cells and epithelial cells in mucosal organs outside the intestine, such as the lung (32). Secondly, fruits, vegetables, nuts, tea, and coffee are rich sources of polyphenols, renowned for their potent antioxidant properties. These bioactive compounds hold the potential to ameliorate cellular injury instigated by oxidative stress by neutralizing free radicals. Moreover, they are implicated in the modulation of DNA methylation, histone modifications, and microRNAs (33). Furthermore, they have been associated with the induction of apoptosis in cancer cells, exerting influence on caspase cascade reactions and pathways such as death receptor 5/p53 (33). Thirdly, limiting the consumption of red and processed meats may attenuate systemic chronic inflammatory responses, thereby potentially reducing the incidence of lung cancer (34).

The present study exhibits a few limitations that warrant thorough discussion. Firstly, the DHQ was completed contemporaneously with the investigation, thereby capturing dietary habits at a specific point in time, which may not adequately represent dietary intake throughout the entirety of a year. Variability in individual dietary patterns, influenced by a multitude of factors, may potentially compromise the precision of the results (35). While baseline dietary assessments capture eating habits at the time of the study, they also reasonably reflect habitual long-term intake patterns based on nutritional epidemiology tenets (36). Hence, the single DHQ measure provided valid representations of participants’ customary diets before and during the study. Secondly, nearly 40,000 participants did not complete a valid DHQ. A significant number of participants did not respond to the DHQ, which may not reflect the true distribution of dietary exposure. Thirdly, while the LLDS demonstrates a certain level of international applicability (15), there exist variations in dietary habits and cultural traditions across different countries and regions. As a result, the LLDS may not entirely capture the genuine dietary status of populations in disparate geographical locales. Fourth, the selection of covariates was informed by pertinent literature and clinical expertise; nevertheless, the possibility of overlooked covariates that could impact the findings cannot be discounted. Fifth, although using the Cox proportional hazards regression model to assess the relationship between LLDS and lung cancer mortality is applicable in our study (37, 38), we acknowledge that deaths from other causes constitute competing risks with lung cancer mortality. Consequently, the use of the Cox proportional hazards regression model may overestimate the cumulative incidence rate.

Conversely, the study also presents several notable strengths. First, this investigation is pioneering in examining the association between LLDS and lung cancer, thereby imbuing its conclusions with considerable significance within the domain of contemporary dietary research. Second, the extensive follow-up period and the inclusion of a large sample size significantly bolstered the statistical power of our study and increased the generalizability of the findings to similar populations. Third, considering that previous studies have shown marital status may influence lung cancer risk (39), we categorized marital status into three groups: Married or Living As Married, Widowed/Divorced/Separated, and Never Married, and included it again as a covariate in our analysis. After controlling for all potential confounding factors, the results of multivariate Cox regression analysis showed that LLDS maintained an inverse association with both lung cancer incidence and mortality (Lung cancer incidence: HR_Q4:Q1_ = 0.80, 95% CI = 0.68–0.94, *P* for trend = 0.004; Lung cancer mortality: HR_Q4:Q1_ = 0.81, 95% CI = 0.67–0.98, *P* for trend = 0.010; Supplementary Table S4). Fourth, rigorous sensitivity analyses were undertaken, including the exclusion of subjects who developed lung cancer within the initial 2 or 4 years, thus bolstering the reliability of our statistical results. Fifth, based on baseline characteristic analysis and sensitivity analysis, the LLDS and HEI-2015 scoring systems may capture similar dietary quality features to some extent, which indirectly substantiates the effectiveness of LLDS in assessing healthy dietary.

## Conclusion

In conclusion, our findings demonstrate that adherence to the LLDS is associated with a reduced incidence and mortality of lung cancer within U.S. adults aged 55 years and older. Notably, this relationship remains consistent in instances of SCLC. These observations offer significant insights and introduce novel perspectives regarding the prevention of lung cancer. However, it is crucial to acknowledge that the study cohort was exclusively comprised of adult Americans aged 55 years and older. Considering the dietary habit differences that are prevalent among various countries, ethnic groups, and age demographics, it is imperative to conduct further validation of this health-promoting dietary pattern across diverse populations and geographical regions.

## Data Availability

The original contributions presented in the study are included in the article/Supplementary material, further inquiries can be directed to the corresponding author.
