# Supplementary material for: Adherence to lifelines diet is associated with lower lung cancer risk in 98,459 participants aged 55 years and above: a large prospective cohort study
Source: Front Nutr. 2024 Oct 23;11:1463481. doi: 10.3389/fnut.2024.1463481 (PMC11537889; doi:10.3389/fnut.2024.1463481)
Supplement: Supplementary file 1 [file Data_Sheet_1.docx]

**Adherence to Lifelines Diet Is Associated With Lower Lung Cancer Risk in 98,459 Participants Aged 55 Years and Above: A Large Prospective Cohort Study**

Yangpiaoyi Shi ^1, #^, Li Xin ^1, #^, Linglong Peng ^1^, Zhiquan Xu ^1^, Hang Liu ^1^, Qi Wei ^1^, Wanhao Tan ^1^, Yaxu Wang ^1^, Ling Xiang ^2^, Haitao Gu ^1,^ *

1 *Department of* *Gastrointestinal Surgery, The Second Affiliated Hospital of Chongqing Medical University, Chongqing, China.*

2 *Department of Clinical Nutrition, The* *Second Affiliated Hospital of Chongqing Medical University, Chongqing, China.*

* Correspondence authors: Haitao Gu, Department of Gastrointestinal Surgery, The Second Affiliated Hospital of Chongqing Medical University, No.288 Tianwen Avenue, Nan'an District, Chongqing, 400010, China. fax: +86 023 6288 7521. E-mail addresses: [ght302211@cqmu.edu.cn](mailto:306359@hospital.edu.cn)

^#^ These authors contributed equally to this work.

Table of contents

**Supplementary Table 1.** Distribution of covariates with missing data before and after imputation.

**Supplementary Table 2.** Sensitivity analyses on the between LLDS and lung cancer incidence.

**Supplementary Table 3.** Sensitivity analyses on the between LLDS and lung cancer mortality.

**Supplementary Table 4.** Hazard ratios of the association between LLDS and lung cancer risk in the additional model with refined marital status categorization

**Supplementary Table 5.** Stratified analyses on the associations of LLDS and lung cancer risk by smoking status.

**Supplementary figure 1**. The flow chart of identifying eligible subjects. PLCO, Prostate, Lung, Colorectal, and Ovarian; BQ, baseline questionnaire; DHQ, diet history questionnaire.

**Supplementary figure 2.** The timeline and follow-up scheme of our study. Notably, in our study, the baseline point was set at the date of diet history questionnaire completion.

**Supplementary figure 1.** The flow chart of identifying eligible subjects. PLCO, Prostate, Lung, Colorectal, and Ovarian; BQ, baseline questionnaire; DHQ, diet history questionnaire.


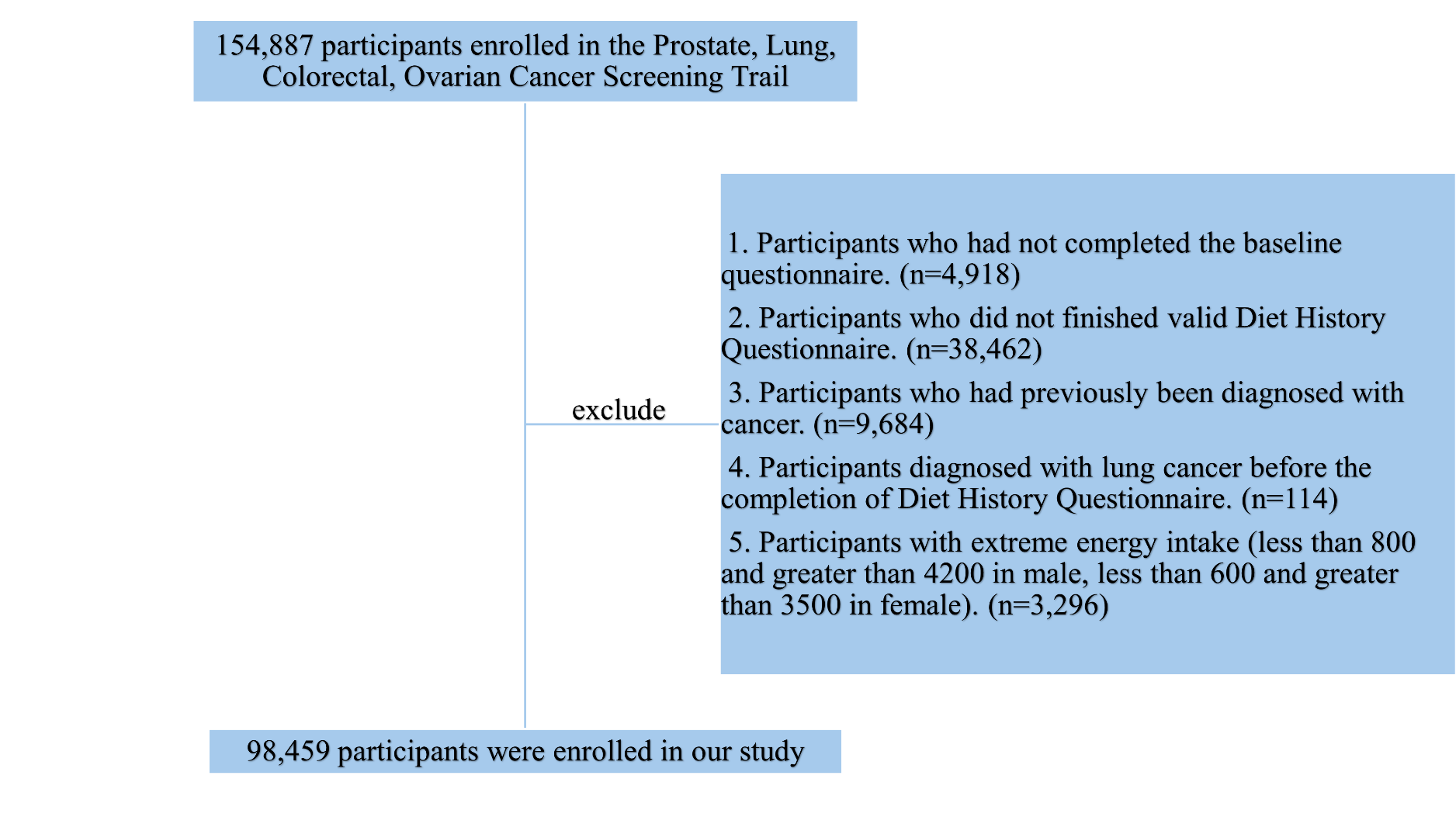


**Supplementary figure 2.** The timeline and follow-up scheme of our study. Notably, in our study, the baseline point was set at the date of diet history questionnaire completion.

**
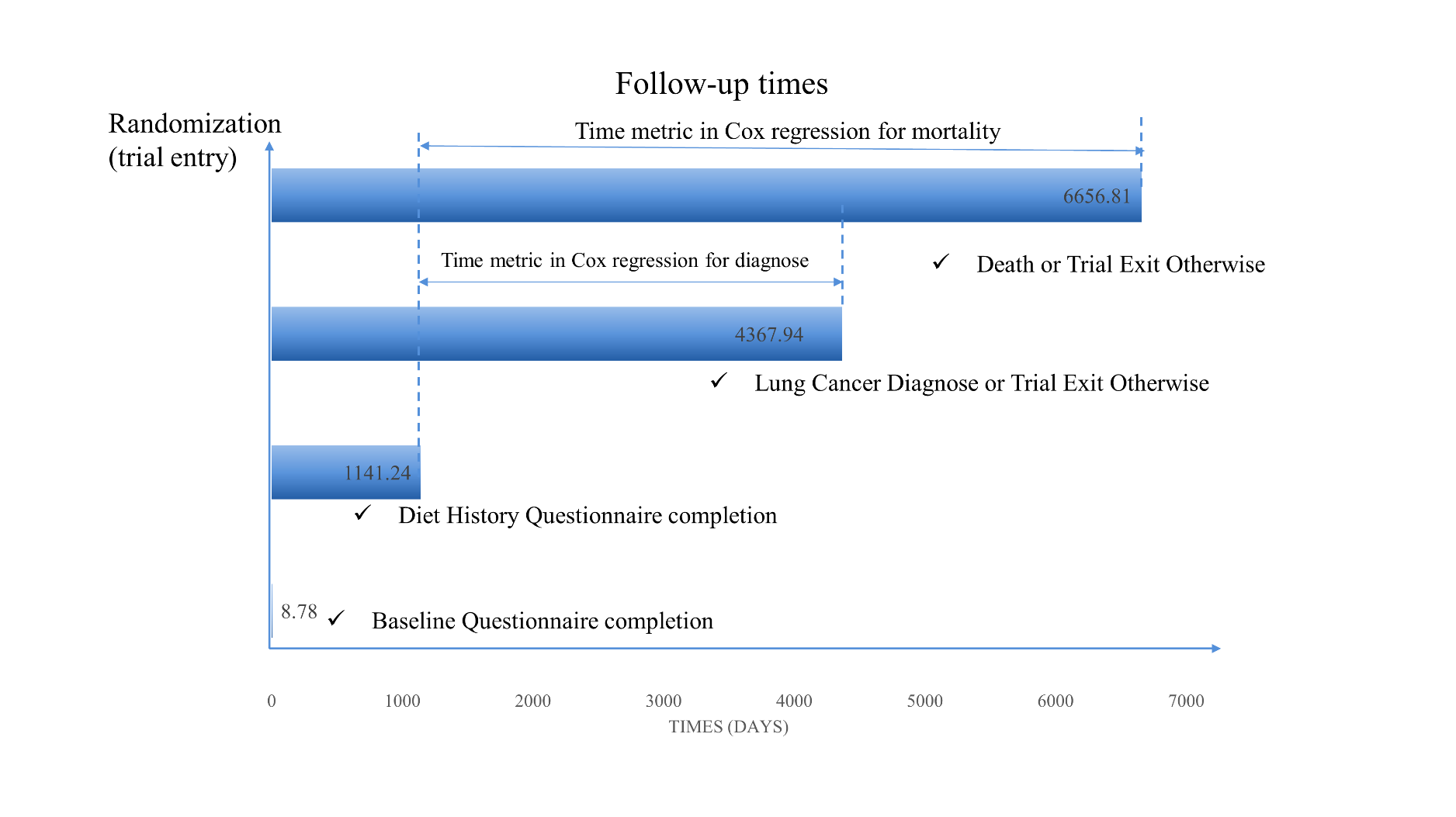
**

**Supplementary Table 1**. Distribution of covariates with missing data before and after imputation

| **Variable** | **Before imputation** | **After imputation** ^a^ | **Number (%) with missing data** |
| --- | --- | --- | --- |
| **Race** |  |  | 34 (0.03%) |
| White | 91187 (92.6%) | 91221 (92.6%) |  |
| Non-White | 7238 (7.4%) | 7238 (7.4%) |  |
| **Marital** |  |  | 185 (0.19%) |
| Married | 77189 (78.5%) | 77374 (78.6%) |  |
| Unmarried | 21085 (21.5%) | 21085 (21.4%) |  |
| **Education** |  |  | 196 (0.20%) |
| College below | 62403 (63.5%) | 62599 (63.6%) |  |
| College graduate | 17353 (17.7%) | 17353 (17.6%) |  |
| Postgraduate | 18507 (18.8%) | 18507 (18.8%) |  |
| **Body mass index (kg/m^2^)** | 27.2±4.8 | 27.2±4.8 | 20 (0.02%) |
| **Smoking status** |  |  | 20 (0.02%) |
| Never | 47213 (48.0%) | 47233 (48.0%) |  |
| Current/Former | 51226 (52.0%) | 51226 (52.0%) |  |
| **Smoking pack-years** | 17.7±26.5 | 17.5±26.4 | 1105 (1.12%) |
| **Family history of lung cancer** |  |  | 757 (0.77%) |
| No | 85088 (87.1%) | 85845 (87.2%) |  |
| Yes | 10266 (10.5%) | 10266 (10.4%) |  |
| Possible | 2348 (2.4%) | 2348 (2.4%) |  |
| **Emphysema history** |  |  | 515 (0.52%) |
| No | 95895 (97.9%) | 96410 (97.9%) |  |
| Yes | 2049 (2.1%) | 2049 (2.1%) |  |
| **Bronchitis history** |  |  | 553 (0.56%) |
| No | 93725 (95.7%) | 94278 (95.8%) |  |
| Yes | 4181 (4.3%) | 4181 (4.2%) |  |
| **History of hypertension** |  |  | 494 (0.50%) |
| No | 66147 (67.5%) | 66641 (67.7%) |  |
| Yes | 31818 (32.5%) | 31818 (32.3%) |  |
| **History of diabetes** |  |  | 520 (0.53%) |
| No | 91470 (93.4%) | 91990 (93.4%) |  |
| Yes | 6469 (6.6%) | 6469 (6.6%) |  |
| **Aspirin use** |  |  | 426 (0.43%) |
| No | 51816 (52.9%) | 52242 (53.1%) |  |
| Yes | 46217 (47.1%) | 46217 (46.9%) |  |

*Values are mean (standard deviation) or counts (percentage) as indicated*

**Supplementary Table 2**. Sensitivity analyses on the between LLDS and lung cancer incidence

| Categories | **participants** | **cases** | HR Quartile 4 vs. Quartile 1 (95% CI) ^a^ | P_-trend_ |
| --- | --- | --- | --- | --- |
| Primary analysis | 98459 | 1642 | 0.80 (0.68, 0.94) | 0.003 |
| Excluded participants with extreme BMI (baseline)^b^ | 96527 | 1609 | 0.80 (0.68, 0.93) | 0.003 |
| Excluded cases observed within the first 2 years of follow-up | 98164 | 1347 | 0.74 (0.62, 0.89) | <0.001 |
| Excluded cases observed within the first 4 years of follow-up | 97836 | 1019 | 0.71 (0.58, 0.87) | <0.001 |
| Excluded participants with respiratory comorbidities ^c^ | 92832 | 1378 | 0.83 (0.70, 0.98) | 0.025 |
| Further adjusted for pack-years of smoking ^d^ | 98459 | 1642 | 0.71 (0.61, 0.84) | 0.003 |
| Employed the HEI-2015 instead of LLDS | 98459 | 1642 | 0.67 (0.58, 0.78) | <0.001 |
| Excluded participants with missing data | 94385 | 1557 | 0.80 (0.68, 0.95) | 0.006 |

*a:* *Hazard ratios were adjusted for age (continuous), sex (male, female), race (white, non-white), education levels (college below, college graduate, postgraduate) , marital status (no, yes), smoking status (never, current/former), pack-years(continuous), alcohol drinking status (no, yes), BMI (continuous), randomization arm (intervention group, control group), family history of lung cancer (no, yes, possibly), history of hypertension (no, yes), history of diabetes (no, yes), history of chronic bronchitis (no, yes), history of emphysema (no, yes) and aspirin use (no, yes).*

*b: The extreme BMI (baseline) was defined as top 1% or bottom 1% in the included population.*

*c: The respiratory comorbidities including chronic bronchitis and emphysema.*

*d: The pack-years of smoking (continuous) was used in the fully adjusted model instead of cigarettes smoked per day (0, 1-20, >20).*

**Supplementary Table 3**. Sensitivity analyses on the between LLDS and lung cancer mortality

| Categories | **participants** | **cases** | HR Quartile 4 vs. Quartile 1 (95% CI) ^a^ | P_-trend_ |
| --- | --- | --- | --- | --- |
| Primary analysis | 98459 | 1172 | 0.81 (0.67, 0.98) | 0.009 |
| Excluded participants with extreme BMI (baseline)^b^ | 96527 | 1147 | 0.79 (0.66, 0.96) | 0.006 |
| Excluded cases observed within the first 2 years of follow-up | 98346 | 1077 | 0.79 (0.65, 0.96) | 0.010 |
| Excluded cases observed within the first 4 years of follow-up | 98160 | 873 | 0.78 (0.63, 0.97) | 0.011 |
| Excluded participants with respiratory comorbidities ^c^ | 92832 | 987 | 0.82 (0.67, 1.00) | 0.032 |
| Further adjusted for pack-years of smoking ^d^ | 98459 | 1172 | 0.72 (0.60, 0.87) | 0.008 |
| Employed the HEI-2015 instead of LLDS | 98459 | 1172 | 0.68 (0.57, 0.80) | <0.001 |
| Excluded participants with missing data | 94385 | 1116 | 0.80 (0.66, 0.97) | 0.011 |

*a: Hazard ratios were adjusted for age (continuous), sex (male, female), race (white, non-white), education levels (college below, college graduate, postgraduate) , marital status (no, yes), smoking status (never, current/former), pack-years(continuous), alcohol drinking status (no, yes), BMI (continuous), randomization arm (intervention group, control group), family history of lung cancer (no, yes, possibly), history of hypertension (no, yes), history of diabetes (no, yes), history of chronic bronchitis (no, yes), history of emphysema (no, yes) and aspirin use (no, yes).*

*b: The extreme BMI (baseline) was defined as top 1% or bottom 1% in the included population.*

*c: The respiratory comorbidities including chronic bronchitis and emphysema.*

*d: The pack-years of smoking (continuous) was used in the fully adjusted model instead of cigarettes smoked per day (0, 1-20, >20).*

**Supplementary Table 4**. Hazard ratios of the association between LLDS and lung cancer risk in the additional model with refined marital status categorization

| Quartiles of LLDS | Cases | Person‐years | Incidence rate per 100 person‐years (95% confidence interval) | Hazard ratio (95% confidence interval) by LLDS | | |
| --- | --- | --- | --- | --- | --- | --- |
|  |  |  |  | Unadjusted | Model 1^a^ | Model 2^b^ |
| **Lung cancer incidence** |  |  |  |  |  |  |
| Quartile 1 | 518 | 214401.6 | 0.24 (0.22, 0.26) | 1.00 (Reference) | 1.00 (Reference) | 1.00 (Reference) |
| Quartile 2 | 485 | 240081.7 | 0.20 (0.18, 0.22) | 0.83 (0.74, 0.94) | 0.89 (0.78, 1.00) | 0.95 (0.84, 1.07) |
| Quartile 3 | 390 | 231673.8 | 0.17 (0.15, 0.19) | 0.69 (0.61, 0.79) | 0.77 (0.67, 0.88) | 0.88 (0.76, 1.00) |
| Quartile 4 | 249 | 183650.8 | 0.14 (0.12, 0.15) | 0.56 (0.48, 0.65) | 0.66 (0.57, 0.78) | 0.80 (0.68, 0.94) |
| P for trend |  |  |  | <0.001 | <0.001 | 0.004 |
| **Lung cancer mortality** |  |  |  |  |  |  |
| Quartile 1 | 382 | 360522.4 | 0.11 (0.10, 0.12) | 1.00 (Reference) | 1.00 (Reference) | 1.00 (Reference) |
| Quartile 2 | 343 | 408370.2 | 0.08 (0.08, 0.09) | 0.80 (0.69, 0.93) | 0.86 (0.74, 1.00) | 0.92 (0.79, 1.07) |
| Quartile 3 | 268 | 397223.4 | 0.07 (0.06, 0.08) | 0.65 (0.55, 0.76) | 0.74 (0.63, 0.86) | 0.84 (0.71, 0.98) |
| Quartile 4 | 179 | 320693.4 | 0.06 (0.05, 0.06) | 0.54 (0.45, 0.65) | 0.67 (0.56, 0.81) | 0.81 (0.67, 0.98) |
| P for trend |  |  |  | <0.001 | <0.001 | 0.010 |

*a: Model 1 was controlled with age (continuous), sex (male, female), race (white, non-white), education levels (college below, college graduate, postgraduate) and marital status (Married or Living As Married, Widowed/Divorced/Separated, and Never Married).*

*b: Model2 was additionally controlled with smoking status (never, current/former), pack-years(continuous), alcohol drinking status (no, yes), BMI (continuous), randomization arm (intervention group, control group), family history of lung cancer (no, yes, possibly), history of hypertension (no, yes), history of diabetes (no, yes), history of chronic bronchitis (no, yes), history of emphysema (no, yes) and aspirin use (no, yes).*

**Supplementary Table 5**. Stratified analyses on the associations of LLDS and lung cancer risk by refined smoking status

| **Outcome** | **Stratified variable** | **Cases** | **Person-years** | **HR_Quartile 4 vs._ _Quartile 1_(95%)** | **P_interaction_** |
| --- | --- | --- | --- | --- | --- |
| Lung cancer incidence | Smoking status |  |  |  | 0.588 |
|  | No | 138 | 424452.3 | 1.37(0.79, 2.39) |  |
|  | Current | 648 | 74638.7 | 1.03(0.78, 1.36) |  |
|  | Former | 856 | 370716.9 | 0.87(0.70, 1.09) |  |
| Lung cancer mortality | Smoking status |  |  |  | 0.790 |
|  | No | 90 | 734811.8 | 1.16(0.59, 2.27) |  |
|  | Current | 457 | 122073.1 | 0.85(0.60, 1.19) |  |
|  | Former | 625 | 629924.5 | 1.01(0.79, 1.30) |  |

*For LLDS, hazard ratios were adjusted for age (continuous), sex (male, female), race (white, non-white), education levels (college below, college graduate, postgraduate) , marital status (no, yes), smoking status (never, current, former), pack-years(continuous), alcohol drinking status (no, yes), BMI (continuous), randomization arm (intervention group, control group), family history of lung cancer (no, yes, possibly), history of hypertension (no, yes), history of diabetes (no, yes), history of chronic bronchitis (no, yes), history of emphysema (no, yes) and aspirin use (no, yes).*
